# Teaching alcohol and smoking counselling in times of COVID-19 to 6^th^-semester medical students: experiences with a digital-only and a blended learning teaching approach using role-play and feedback

**DOI:** 10.3205/zma001513

**Published:** 2021-11-15

**Authors:** Elena Tiedemann, Anne Simmenroth

**Affiliations:** 1 University Hospital Würzburg, Department of General Practice, Würzburg, Germany

**Keywords:** e-learning, prevention, role-play, reflection, alcohol consumption, smoking, counselling

## Abstract

**Objectives: **Digital teaching formats have seen increased use, and not just since the beginning of the pandemic. They can also be used to teach cognitive, practical and communicative learning objectives effectively. We describe the implementation of an online-only course on alcohol/smoking counselling in the COVID-19 summer semester (SS) 2020 and an inverted classroom (IC) concept in the winter semester (WS) 2020/21 at the University Hospital of Würzburg.

**Methodology: **The interdisciplinary subject of “prevention” teaches students about high-risk alcohol consumption/smoking and how to conduct a brief verbal intervention. All 143 (SS) and 131 (WS) 6^th^-semester medical students completed a 90-minute course: module 1 included a Prezi^®^ presentation on alcohol/smoking basics. Module 2 introduced a counselling concept (alcohol or smoking) online or classroom-based (WS only), depending on the participants’ choice. In the online practical component, each student created a counselling video and reflections at home, and later received written feedback from lecturers/tutors. Supervised role-playing was used in the classroom-based format in the WS. There were 2 exam questions on module 1 at the end of each semester.

**Results: **The students surveyed (11%) were satisfied with module 1. Practical exercises and feedback received praise in the evaluation of the classroom-based format (response: 97%). It was not possible for all students to perform counselling due to time constraints. A majority of participants filmed live role-playing in the online practical component. The exam questions were answered correctly by 31% (SS) and 36% (WS) respectively.

**Conclusions: **Counselling can also be taught digitally: creating one’s own videos with delayed written feedback is an innovative form of teaching. We are now aiming for a mix of both aspects as an IC with 90 minutes of classroom-based practical exercises.

## Zusammenfassung

**Zielsetzung: **Nicht erst seit Beginn der Pandemie werden digitale Lehrformate verstärkt eingesetzt. Kognitive, praktische und kommunikative Lernziele können auch so effektiv vermittelt werden. Wir beschreiben die Umsetzung eines reinen Online-Kurses zur Alkohol-/Raucherberatung im COVID-19-Sommersemester (SS) 2020 und ein Inverted Classroom (IC)-Konzept im Wintersemester (WS) 2020/21 am Universitätsklinikum Würzburg.

**Methodik: **Im Querschnittsfach „Prävention“ wird Wissen zum riskanten Alkoholkonsum/Rauchen und das Durchführen einer verbalen Kurzintervention unterrichtet. Alle 143 (SS) bzw. 131 (WS) Medizinstudierenden des 6. Semesters durchliefen einen auf 90 Minuten angelegten Kurs: Modul 1 umfasste eine Prezi^®^-Präsentation über Grundlagen zu Alkohol/Rauchen. In Modul 2 wurde je nach Wahl ein Beratungskonzept (Alkohol oder Rauchen) online oder in Präsenz (nur WS) vorgestellt. Im Online-Praxisteil erstellte jeder Studierende zu Hause ein Beratungsvideo und eine Reflexion, später gab es schriftliches Feedback von Dozierenden/Tutor*innen. In der Präsenzform im WS wurden Rollenspiele unter Supervision durchgeführt. Jeweils am Semesterende folgten 2 Klausurfragen zu Modul 1.

**Ergebnisse: **Die befragten Studierenden (11%) zeigten sich zufrieden mit Modul 1. In der Evaluation der Präsenzform (Response: 97%) wurden praktisches Üben und Feedback gelobt. Aus Zeitgründen führten nicht alle eine Beratung durch. Im Online-Praxisteil drehte die Mehrheit ein Rollenspiel live. Die Klausurfragen lösten 31% (SS) bzw. 36% (WS) richtig.

**Schlussfolgerung:** Beratung kann auch digital gelehrt werden: Das Drehen eigener Videos mit zeitlich versetztem schriftlichem Feedback ist eine innovative Lehrform. Wir streben nun einen Mix beider Themen als IC mit 90 Minuten praktischen Übungen in Präsenz an.

## Introduction

It is not just since the beginning of the COVID-19 pandemic that there has been increased discussion about the use of digital teaching formats such as blended learning (BL) [1], [2]. The inverted classroom (IC), is particularly well suited for this type of teaching: after an initial (online) self-learning phase, a classroom event [2] (or an online event) [3]) is then held with students who have been prepared for the subject. Even if studies in this area are still heterogeneous, initial research suggests that e-learning can be at least as effective [4] and BL or IC with a classroom-based component can even be more successful than traditional forms of teaching [5], [6]. Even communication skills seem to be taught effectively through digital means [7]. These are needed, for example, in addiction-related counselling within the framework of the interdisciplinary subject of prevention. Digital teaching is already taking place in this area as well [8], [9].

Irrespective of the form of teaching, counselling patients who smoke or drink irresponsibly is still an underrepresented field in medical studies [10], even though brief interventions are considered to be effective [11], [12]. A course on both subjects was therefore piloted for the first time at the University Hospital of Würzburg (UKW) in the summer semester (SS) 2020 and held again in the winter semester (WS) 2020/21. 

The main learning objectives of the course were to teach the medical and epidemiological basics of high-risk alcohol consumption/smoking and to conduct a brief verbal intervention (see table 1 (Tab. 1)). These learning objectives are also reflected in the National Competence-Based Catalogue of Learning Objectives in Medicine (Nationaler Kompetenzbasierter Lernzielkatalog Medizin – NKLM; [http://www.nklm.de]), according to which medical students should be able to “name addiction-specific risks and communicate them in counselling” (VIII.4-04.3.3.) or “conduct counselling and, if necessary, interventions to change behaviour and lifestyle based on essential knowledge” (VIII.2-04.3.3.). The foundation of knowledge required for this should be laid in the first four semesters; we offer the course described below in the 6^th^ semester. 

The article describes experiences gained from the implementation of an online-only module on alcohol/smoking counselling in the Covid summer semester 2020 and a complementary inverted classroom concept with a classroom-based component in the winter semester 2020/21 at the University Hospital of Würzburg.

## Project description

### Participants

In the SS, all 143 6^th^-semester students took part in the interdisciplinary course on prevention and health promotion (Q10), in the WS 131 (62% female in each case).

Q10 comprises 2 semester hours per week with a total of 16 lectures covering a wide range of topics (including prevention of mental illness in adulthood, colorectal cancer screening, prenatal care, tertiary prevention etc). In addition, there is a vaccination course with two lectures, ten virtual patient cases and a subsequent classroom-based course. All lectures were made available as PowerPoint presentations set to music during the COVID-19 semesters.

#### Course outline 

**Figure 1:**
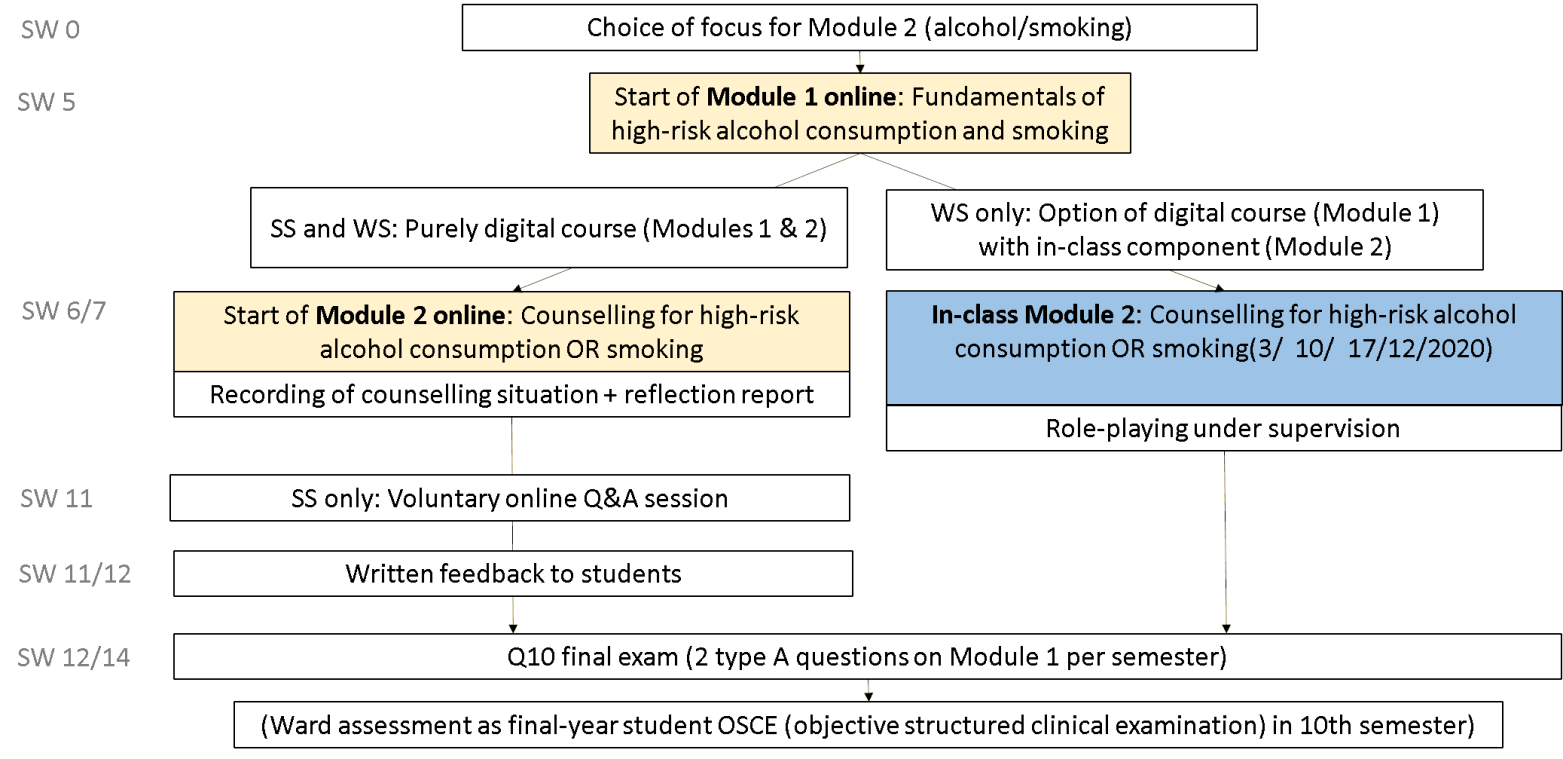
Course schedule (SW: semester week)

Our course (see figure 1 (Fig. 1)) comprised a theoretical part on high-risk alcohol consumption and nicotine addiction (module 1) and a practical component with exercises on motivational interviewing skills (module 2). It was possible to choose between counselling for high-risk alcohol consumption and counselling for smoking in module 2.

The five learning objectives of the course are presented in table 1 (Tab. 1) based on the concept of constructive alignment [13]. The achievement of the learning objectives in module 1 was tested using summative assessment in the final exam. Formative feedback on role-playing in a counselling situation was given in module 2.

Initially, it was planned to teach module 1 online in the form of an inverted classroom [5] with a classroom-based component and to follow up with module 2 as a classroom-based seminar with role-playing. Owing to the COVID-19 pandemic, module 2 also had to be taught completely online in the SS; in the WS, students could choose between attendance or online. 

#### Module 1: Theory of smoking and alcohol consumption

Module 1 comprised 160 Prezi^®^ slides (half on alcohol and half on smoking), a podcast of a patient with alcohol dependence (3 min) [14] and short explanatory videos [15], [16], [17]. Besides a chronological view through “zooming in” on individual topics, Prezi allows a self-selected sequence of events [18]. The premium version used here also allows usage data (dwell time) to be recorded. Activating elements were built into the Prezi presentation in places (“guestimate questions”, reflections on participants’ own alcohol consumption), which were not recorded. Module 1 ended with a quality control evaluation of the module (6 closed, 4 open questions) on EvaSys, which asked, for example, about the evaluation of the module, the time taken to complete it, and suggestions for improvement. EvaSys is evaluation software that can process both online and paper questionnaires and has been used as standard at the University of Würzburg for a considerable time [19].

#### Module 2: Counselling for high-risk alcohol consumption/smoking

##### Online

The Prezi presentation on smoking counselling featured the WHO guideline on counselling according to the “5 as” [20] and a sample counselling video (15 min) [21]. Similarly, counselling for high-risk alcohol consumption featured slides and a video demonstration (5 min) [22]. Both Prezi presentations ended with the assignment for the practical section and an evaluation (WS only). The practical part – as a substitute for role-playing in the course – required a video to be filmed of a student trainee (max. duration 10 min) and a reflection report to be submitted (see table 2 (Tab. 2)). The person in the patient role (fellow student/family member) was expected to give structured feedback. Role scripts, feedback guidance and interview guidelines were provided for both counselling subjects (templates: [23], [24]). 

All students were also given personal written feedback on the counselling video and the reflection report (see table 1 (Tab. 1)), in the SS by course lecturers, in the WS some of the feedback was also written by 2 tutors under supervision.

##### Classroom attendance (WS only)

The theory for the 60-minute classroom-based module in the winter semester was presented by 1-2 lecturers with 15 PowerPoint slides, similar to the online Prezi presentation. The lecturers then worked with two tutors to supervise role-playing in groups of three (student, patient, observer), which was carried out observing minimum distance requirements and wearing a mask. Role scripts and interview guides were identical to the material from the video production. There were 40 minutes available for 2-3 interviews with feedback. A short paper-based evaluation followed at the end.

##### Written exam

The final exam of the Q10 Prevention includes 20 MC questions every semester dealing with very different subjects due to the broad spectrum of Q10. Two questions of the 20 questions asked each semester focus on the learning objectives of Module 1 of the course described (see the attachment 1 for an example of exam questions).

##### Training of the teaching staff

The course teaching staff were trained in advance. The tutors (medical students of higher semesters) took part in a two-hour training session with a focus on giving feedback using counselling videos and were familiarised with the procedure of the classroom-based course. Training was led by the two authors of the article.

The course lecturers were doctors and psychologists from the Department of General Practice. Lecturers teaching in the classroom-based course had previously observed the class and participated in a half-hour introduction via Zoom. Lecturers were sent the course materials for the evaluation of the videos of the online component by e-mail and were able to contact experienced course lecturers if they had any questions. The counselling videos submitted were viewed by the lecturers, most of whom had also taught the course in the previous semester (before the pandemic).

##### Course organisation and data privacy

The course materials were developed by lecturers and doctoral students after studying the literature and piloted in WS 18/19 (smoking) [25] and WS 19/20 (alcohol).

The materials were uploaded to WueCampus learning platform, which is commonly used at the UKW, and the videos and reflection reports were uploaded by the students to GigaMove [26]. The web application, which is free of charge for members of the university, allows a file of up to 2 GB to be uploaded with password protection. The files submitted were stored on a password-protected drive of the UKW.

A privacy policy was uploaded in the Prezi presentation and in the WueCampus room. It informed students that the transmitted data (including audio and video data) would be processed exclusively for the purposes of the course “Prevention – General Medicine Seminar” (with authorisation in accordance with article 6 (1) (a), (b) and (c) GDPR). It was stated that the data would be stored in accordance with the retention period for examination documents (2 years) and then destroyed, and that no data would be disclosed to third parties. When submitting the counselling video, the students declared that they had taken note of the privacy policy and consented to it. 

## Results

### Module 1

On average, Module 1 was given a rating of 1.7 (SS) and 1.3 (WS), with only 11% (n=16/15) of students giving a rating in each case. In the free text answers in the SS, the activating elements in the form of videos, Prezi and the design of the module were highlighted positively, as was also the case in the WS; in addition, Prezi was perceived to be more varied in comparison with PowerPoint. According to Prezi Analytics, students spent a total of approx. 140 (SS) and 107 (WS) hours on Module 1, which, based on the number of students in the relevant semester, corresponds to working time of 59 and 49 minutes per student. It should be noted here that Prezi Analytics stops counting if there is at least one minute of inactivity, and so the time spent playing the videos was probably not recorded in full. According to information provided by the students themselves in the evaluation, the median working time was 60 (20-150 min; SS) and 70 (35-200 min; WS) minutes respectively.

#### Module 2

The majority of students chose “high-risk alcohol consumption” for counselling (SS: 64%; WS: 61%). In the WS, online and classroom-based learning were equally popular (classroom-based: 49%, online: 51%).

Analyses of module 2 can be found in table 3 (Tab. 3). Due to the low response rate (n=3 per topic), the evaluation of the online part was not evaluated; 65 students participated in the evaluation of classroom-based learning in the WS (response rate: 97%).

The size of groups in the 5 classroom-based courses ranged between 11 and 19 students (coronavirus-related restriction to 28 people). A large part of the respondents agreed or strongly agreed with the statement that they would be confident to have a short counselling session with patients who smoke (88%, n=24) or drink alcohol at a high-risk level (83%, n=40). Role-playing was positively highlighted in the free-text answers of the evaluation, although there was also criticism that the time was too short and not everyone in the group of three could take on the role of student trainee. There was also a request for video examples.

In the online part, almost all students performed role-playing in a live situation with a person of the same age, probably a fellow student or flatmate. Five (SS) and 15 (WS) students conducted counselling via Skype. Videos were submitted with one exception in each semester (audio). Most counselling sessions followed the script of the patient role, occasionally other counselling sessions (own role, real counselling event) were conducted. 

The self-made videos lasted 4-20 minutes, with some of the feedback also being recorded. The amount of work required for the assessment of a video by the lecturers was measured by the length of the video; in addition, an estimated minimum of 10 minutes was required to formulate the bullet-point feedback and to read the reflection report. According to this estimate, at least 20 hours were needed to write the feedbacks in the winter semester and twice as many hours in the summer semester. 

#### Written exam

The only two questions in the MC exam of the QB targeting factual knowledge cannot be used to evaluate the teaching concept presented here, which aims at the acquisition of skills, but should nevertheless be briefly presented here. An average total of 18 questions out of the 20 MC questions were answered correctly in both semesters. The mean difficulty of all questions was p=0.85, the discriminatory power was low (ritc=0.00-0.35).

The two questions targeting our module (see attachment 1 ) were the most difficult in the exam in both the SS (n=142) and the WS (n=129), with one exception: only a third answered both correctly (SS: 31%, WS: 36%), while a further 47% (SS) and 36% (WS) at least one question, and around a quarter no question (SS: 22%, WS: 27%). In the SS, the questions on standard glasses (learning objective 1; 53.5%) and on stopping smoking during pregnancy (learning objective 1; 55.6%) were answered correctly by half of the students in each case. In the WS, also slightly more than half of the students solved the question on alcohol screening with the CAGE questionnaire [27] (learning objective 2; 51.9%) and on the transtheoretical model of behavioural change [28] (learning objective 3; 57.4%). 

## Discussion

Teaching under pandemic conditions is especially challenging in the area of communication. Our surveys from two semesters have shown that knowledge and communicative competence on the topic of “high-risk alcohol consumption” and “smoker counselling” can also be implemented well using the inverted classroom concept or asynchronously using purely electronic methods. The main learning objectives were to teach the medical and epidemiological basics of high-risk alcohol consumption/smoking and to conduct a brief intervention.

The sole indicator for the achievement of the learning objectives in Module 1 were two questions in the final exam, which were answered correctly by only one third of the students in both semesters. One possible explanation for the rather poor performance could be insufficient take-up of module 1. This is contradicted by the fact that, according to Prezi Analytics, students spent an average of at least three quarters of an hour on module 1. However, this value is only a rough estimate. It is unclear whether some students made extensive use of the module and others hardly used it at all, as suggested by the wide variations in self-reported completion times in the evaluation. It might therefore be possible that those who answered the exam questions correctly invested more time in module 1. An alternative explanation is that the questions were too specific, so that even if the module was studied in depth, the questions were too difficult and therefore could not adequately test whether the learning objective had been achieved.

The main learning objective of module 2 was for each student to conduct a counselling session on smoking or alcohol themselves in the form of a brief intervention. This was assured for the online version, as each student had to submit their own video. In the classroom-based version, 60 minutes were not always enough for three role plays in the small group. Although the majority of students in the classroom-based course agreed with the statement that they had the confidence to conduct a counselling interview, this learning objective was not achieved by all of them. 

Our online learning enabled multiple reflection on the counselling interview. Students reflected in writing, ideally received verbal feedback from their role-play partner and again written feedback from lecturers/tutors. The videos showed that many students had made their own notes and had studied the material thoroughly beforehand. Exploring one's own strengths/weaknesses on the basis of videos with the help of open questions is a suitable self-assessment method [29]. As Hammoud et al. recommend on the basis of their review, video-based feedback should ideally combine students' self-assessment with feedback from experts [30].

In the WS, it was evident that the classroom-based programme was just as popular. Here students were particularly satisfied with the personal feedback in the role-playing, including from tutors, which is consistent with the literature [31]. The personal communication with the students in the fixed framework of the classroom-based course, in contrast to the online variant, ensured that the feedback reached the students directly and was actually given. Accordingly, one person in the group of three was only ever responsible for giving feedback. It is unclear whether the participants of the online course had actually all received feedback from their role-playing partners, and whether all of them actually reflected on their counselling video after receiving feedback from the experts. 

### Limitations

Participation in the online evaluations was low, which could be related to the many additional online surveys during the electronic semesters and also affected many other teaching events in Würzburg. According to information from the Dean of Studies, the response to the evaluations in the semester in question was at an “historic low” of 11%. A similar situation was reported in Göttingen, for example, where only one in five students took part in the evaluation in the Covid summer semester of 2020, compared to 50% in the classroom-based semester [32]. Another reason could be that evaluation was not asked for separately, but appeared online directly at the end of the Prezi presentation. In the classroom-based course, almost all students took part in the evaluation after being approached personally. 

Irrespective of the teaching format, learning success in relation to counselling skills could not be objectively measured after conducting a counselling session; a PJ maturity OSCE (objective structured clinical examination) will not take place until 2022/2023 for the cohort of students investigated. Nevertheless, we suspect learning success from our course as it includes elements that have proven effective in increasing counselling skills at other sites (concept 5 as/transtheoretical model of behavioural change, activating methods, feedback) [33], [34].

#### Outlook

It has been shown that, as a rule, 60 minutes is not sufficient to perform role-playing with feedback three times. It is therefore planned to offer Module 2 from the winter semester 2021/22 for all students as a 90-minute classroom-based event on both main topics (alcohol and smoking) at the same time with role-playing. Module 1 is to be preceded by an inverted classroom, and counselling videos are to be watched in advance of the course, as was also requested in the classroom-based course. If a classroom-based module is not possible for pandemic reasons, it will be possible to switch back to a purely online course at any time, so that every student has the opportunity to practise. 

The simultaneous teaching of both main areas ties in with the observation of the review by Hauer et al., according to which courses on counselling often only focus on one area (most frequently alcohol or smoking counselling), but patients often come with different or multiple reasons for counselling and prospective doctors should be prepared for as many of them as possible [34]. It is therefore planned to extend and apply the 5 A model in the role-playing to other counselling situations such as obesity in the future.

## Conclusion

Alcohol and smoking counselling can be taught purely asynchronously electronically or as an inverted classroom with a classroom-based component.

- Online components are particularly suitable for the presentation of videos.
- Creating your own videos with delayed written feedback is an innovative alternative form of teaching in a pandemic.
- Feedback – in that case verbal – can be given more directly in the classroom-based course.

The online module 1 will be extended to include example counselling videos to combine the benefits, and the classroom-based course for module 2 will be extended to 90 minutes.

## Competing interests

The authors declare that they have no competing interests. 

## Supplementary Material

Exam questions

## Figures and Tables

**Table 1:**
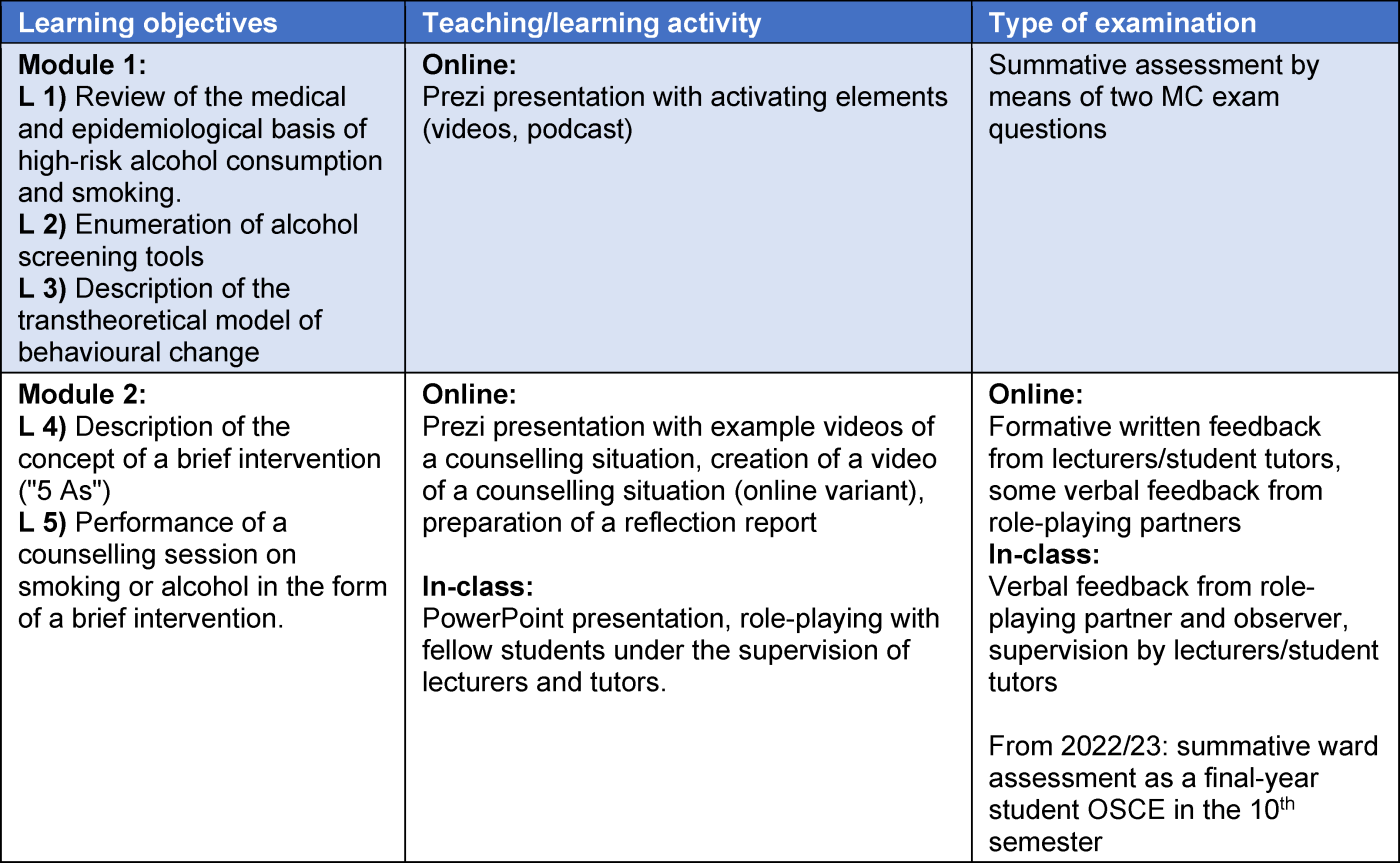
Learning objectives (L) of Modules 1 and 2 based on the concept of constructive alignment [13]

**Table 2:**
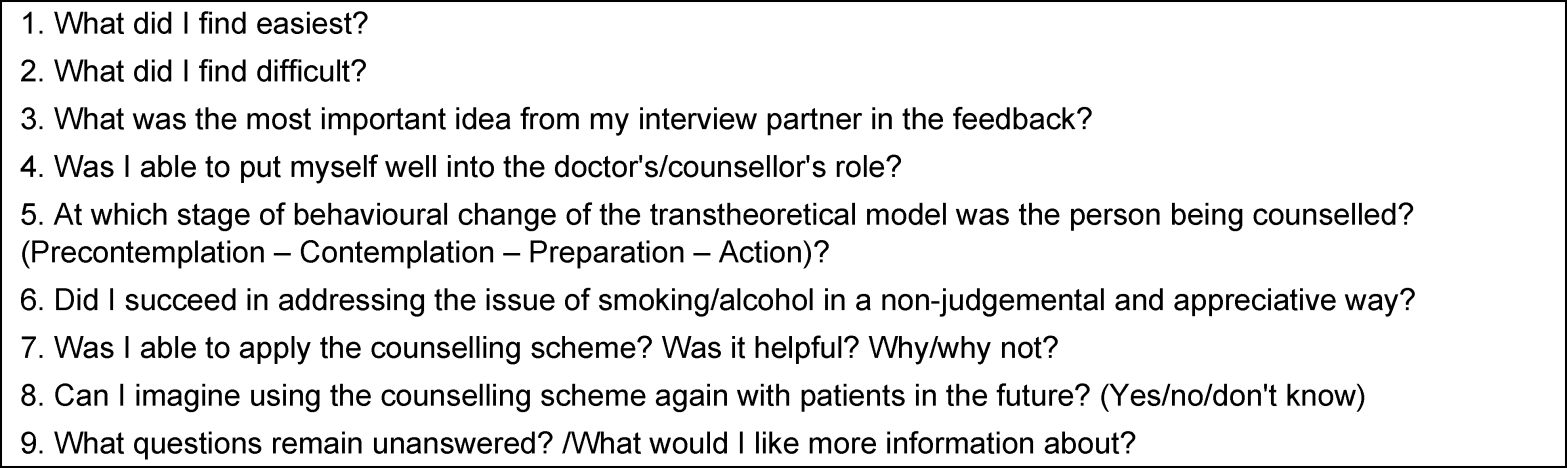
Questions for the reflection report

**Table 3:**
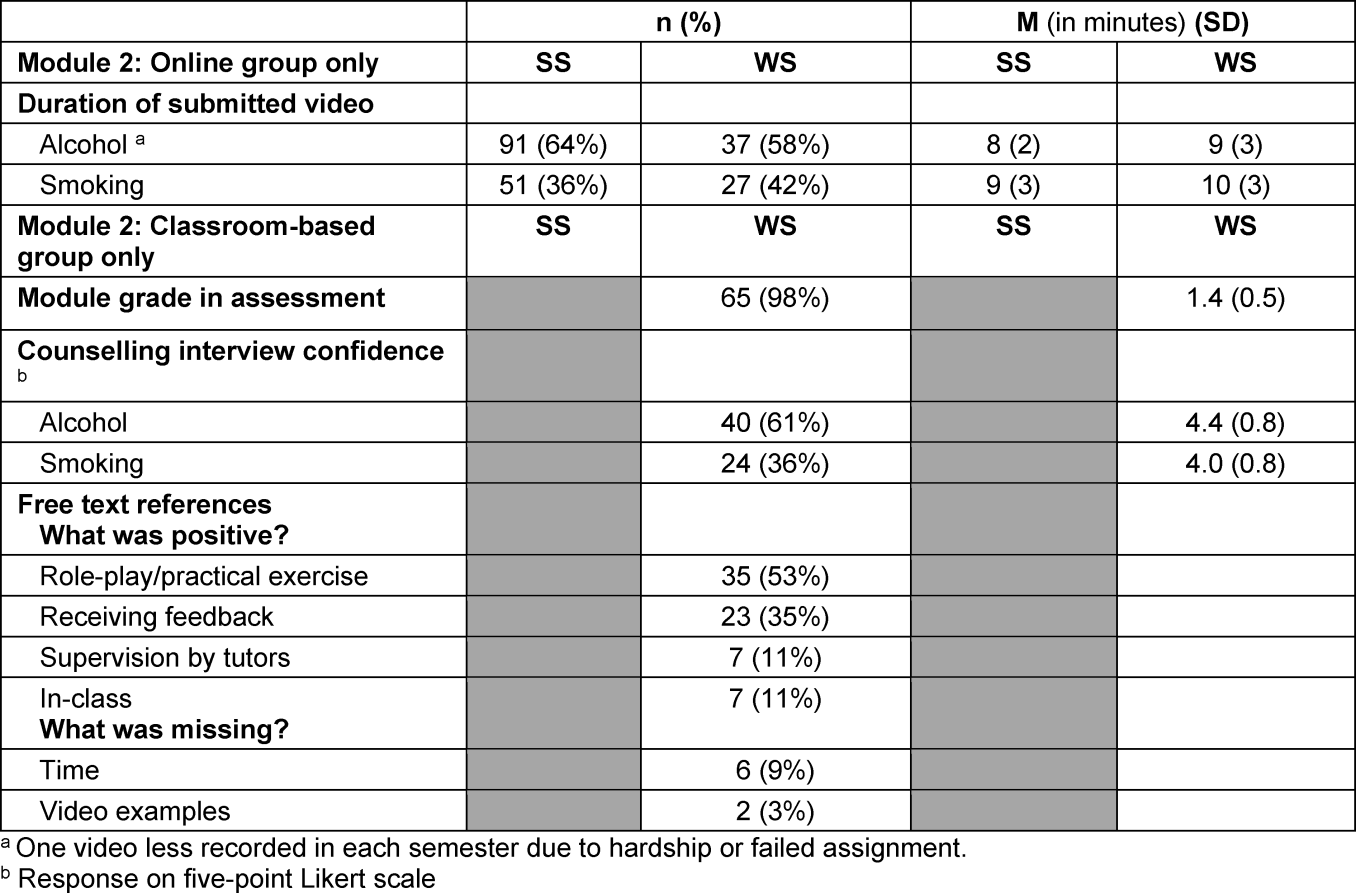
Results from Module 2 in the summer semester (SS; online) and winter semester (WS; online and classroom-based)

## References

[1] Skulmowski A, Rey GD (2020). COVID-19 as an accelerator for digitalization at a German university: Establishing hybrid campuses in times of crisis. Hum Behav Emerg Technol.

[2] Kuhn S, Frankenhauser S, Tolks D (2018). Digital learning and teaching in medical education: Already there or still at the beginning?. Bundesgesundheitsblatt Gesundheitsforschung Gesundheitsschutz.

[3] Huber J, Witti M, Schunk M, Fischer MR, Tolks D (2021). The use of the online Inverted Classroom Model for digital teaching with gamification in medical studies. GMS J Med Educ.

[4] Pei L, Wu H (2019). Does online learning work better than offline learning in undergraduate medical education? A systematic review and meta-analysis. Med Educ Online.

[5] Hew KF, Lo CK (2018). Flipped classroom improves student learning in health professions education: a meta-analysis. BMC Med Educ.

[6] Liu Q, Peng W, Zhang F, Hu R, Li Y, Yan W (2016). The Effectiveness of Blended Learning in Health Professions: Systematic Review and Meta-Analysis. J Med Internet Res.

[7] Kyaw BM, Posadzki P, Paddock S, Car J, Campbell J, Tudor Car L (2019). Effectiveness of Digital Education on Communication Skills Among Medical Students: Systematic Review and Meta-Analysis by the Digital Health Education Collaboration. J Med Internet Res.

[8] Simansalam S, Brewster JM, Nik Mohamed MH (2015). Training Malaysian Pharmacy Undergraduates with Knowledge and Skills on Smoking Cessation. Am J Pharm Educ.

[9] Lee JD, Triola M, Gillespie C, Gourevitch MN, Hanley K, Truncali A, Zabar S, Kalet Al (2008). Working with Patients with Alcohol Problems: A Controlled Trial of the Impact of a Rich Media Web Module on Medical Student Performance. J Gen Intern Med.

[10] Strobel L, Schneider NK, Krampe H, Beißbarth T, Pukrop T, Anders S, West R, Aveyard P, Raupach T (2012). German medical students lack knowledge of how to treat smoking and problem drinking. Addicition.

[11] Kaner EF, Beyer FR, Muirhead C, Campbell F, Pienaar ED, Bertholet N, Daeppen JB, Saunders JB, Burnand B (2018). Effectiveness of brief alcohol interventions in primary care populations. Cochrane Database Syst Rev.

[12] Stead LF, Buitrago D, Preciado N, Sanchez G, Hartmann-Boyce J, Lancaster T (2013). Physician advice for smoking cessation. Cochrane Database Syst Rev.

[13] Biggs J (2014). Constructive alignment in university teaching. HERDSA Rev High Educ.

[14] WDR 5 Leonardo (2018). Ein trockener Alkoholiker erzählt.

[15] Alkohol? Kenn dein Limit (2017). Alkohol: Wie viel steckt in einem Standardglas?.

[16] Frädrich S (2013). GedankenTanken. So hörst du sofort mit dem Rauchen auf!.

[17] (2018). Neuroscientifically Challenged. 2-Minute Neuroscience: Alcohol.

[18] Perron BE, Stearns AG (2011). A Review of a Presentation Technology: Prezi. Res Soc Work Pract.

[19] Universität Würzburg Qualitätsmanagement: Software: EvaSys.

[20] World Health Organization (2014). Toolkit for delivering the 5A's and 5R's brief tobacco interventions to TB patients in primary care.

[21] Behavioral Health & Wellness Program (2015). The 5 A's and Tobacco Cessation.

[22] SBIRT Oregon (2010). Brief intervention: "Steve".

[23] SBIRT Oregon (2010). Role play: Jill.

[24] SBIRT Oregon Pocket Card Alcohol.

[25] Lauerer E, Tiedemann E, Polak T, Simmenroth A (2021). Can smoking cessation be taught online? A prospective study comparing e-learning and role-playing in medical education. Int J Med Educ.

[26] RWTH Aachen University Gigamove - RWTH Aachen.

[27] Mayfield D, McLeod G, Hall P (1974). The CAGE questionnaire: validation of a new alcoholism screening instrument. Am J Psychiatry.

[28] Prochaska JO, Velicer WF (1997). The transtheoretical model of health behavior change. Am J Health Promot.

[29] Zick A, Granieri M, Makoul G (2007). First-year medical students' assessment of their own communication skills: A video-based, open-ended approach. Patient Educ Couns.

[30] Hammoud M, Morgan HK, Edwards ME, Lyon JA, White C (2012). Is video review of patient encounters an effective tool for medical student learning? A review of the literature. Adv Med Educ Pract.

[31] Mills JK, Dalleywater WJ, Tischler V (2014). An assessment of student satisfaction with peer teaching of clinical communication skills. BMC Med Educ.

[32] Seifert T, Becker T, Büttcher AF, Herwig N, Raupach T (2021). Restructuring the clinical curriculum at University Medical Center Göttingen: effects of distance teaching on students' satisfaction and learning outcome. GMS J Med Educ.

[33] Hyndman K, Thomas RE, Schira HR, Bradley J, Chachula K, Patterson SK, Compton SM (2019). The Effectiveness of Tobacco Dependence Education in Health Professional Students' Practice: A Systematic Review and Meta-Analysis of Randomized Controlled Trials. Int J Environ Res Public Health.

[34] Hauer KE, Carney PA, Chang A, Satterfield J (2012). Behavior change counseling curricula for medical trainees: a systematic review. Acad Med.

