# Supplementary material for: Teaching alcohol and smoking counselling in times of COVID-19 to 6th-semester medical students: experiences with a digital-only and a blended learning teaching approach using role-play and feedback
Source: GMS J Med Educ. 2021 Nov 15;38(7):Doc117. doi: 10.3205/zma001513 (PMC8675379; doi:10.3205/zma001513)
Supplement: Exam questions [file JME-38-7-117-s-001.pdf]

Anhang: Klausurfragen

Tabelle 1: Modulbezogene Klausurfragen am Semesterende

Sommersemester 2020 (L1 [a])	Wintersemester 2020/21 (L2 & L3 [a])
**[TypA] Welche Aussage zum Alkoholkonsum trifft am ehesten zu?**<br>1. Ein Standardglas Alkohol enthält ca. 20 g reinen Alkohol<br>2. „Risikoarmer Konsum“ bei Männern beinhaltet laut Definition der Deutscher Hauptstelle für Suchtfragen bis zu 3 Standardgetränke pro Trinkgelegenheit.<br>3. Pro Kopf wird in Deutschland ca. 50 Liter reiner Alkohol pro Jahr getrunken.<br>**4. Gefährlicher Konsum betrifft ca. 2% der Deutschen zwischen 18 und 64 Jahren. (richtig)**<br>5. Eine Maß Bier (1 l) enthält ca. 60 g reinen Alkohol.	**[TypA] Herr Müller (60 Jahre, berufstätig) kommt heute in Ihre Hausarztpraxis. Neben Zigaretten gibt Herr Müller an, fast täglich eine Flasche Wein zu konsumieren. Sie vermuten eine Abhängigkeit. Welche der folgenden Fragen gehört nicht zum CAGE-Fragebogen?**<br>**1. Haben Sie wegen des Trinkens einmal Probleme am Arbeitsplatz bekommen? (richtig)**<br>2. Empfinden Sie Schuldgefühle aufgrund Ihres Trinkverhaltens?<br>3. Haben Sie jemals daran gedacht, weniger zu trinken?<br>4. Brauchen Sie morgens nach dem Aufwachen Alkohol, um leistungsfähig zu werden?<br>5. Ärgert Sie die Kritik Ihres Umfelds an Ihrem Alkoholkonsum?
**[TypA] Sie sind Hausärztin/Hausarzt in einer Praxis. Zu Ihnen kommt Frau Schmidt, promovierte Chemikerin mit gutem Einkommen. Vom heutigen Termin erhofft sie sich Hilfe bei einem Rauchstopp, da sie trotz Schwangerschaft (6. Woche) nicht endgültig mit dem Rauchen aufhören kann. Welche Aussage trifft nicht zu?**<br>1. Unabhängig vom Sozialstatus gehört sie damit zu 12 % der Frauen in Deutschland, die während der Schwangerschaft rauchen.<br>2. Unabhängig von der Schwangerschaft und Sozialstatus gehört sie damit zu den 20 % Frauen, die in Deutschland rauchen.<br>3. Im transtheoretischen Modell befindet sie sich damit im Stadium der Vorbereitung.<br>4. Sie gehört damit zu nur weniger als 3 % der Schwangeren mit hohem Sozialstatus, die während der Schwangerschaft rauchen.<br>**5. Um schnell Erfolge beim Rauchstopp zu erzielen, unterstützen Sie den Rauchstopp durch ein Medikament wie z.B. Vareniclin. (richtig)**	**[TypA] Der Test ergibt keinen Hinweis auf eine Abhängigkeit. Laut Trinkmenge weist Herr Müller aber einen gefährlichen Konsum auf, möchte aber an der Trinkmenge in der nächsten Zeit nichts ändern. Damit befindet er sich auf der Stufe der Absichtslosigkeit im Modell von Prochaska und di Clemente.**<br><br>**Was sollten Sie zu diesem Zeitpunkt am ehesten tun?**<br><br>1. Ihm vor allem die Konsequenzen seines derzeitigen Konsums aufzeigen.<br>2. Ihn überreden, jetzt weniger zu trinken.<br>3. Konkrete Ziele zur Trinkmengenreduktion mit ihm vereinbaren.<br>4. Ihn Vorschläge machen lassen, wie er seinen Konsum reduzieren kann<br>5. **Durch Informationen Interesse am Thema Alkoholkonsum wecken. (richtig)**

[a] L: Lernziele gemäß Tabelle 1.

Anhang 1 zu: Simmenroth A, Tiedemann E. *Teaching alcohol and smoking counselling in times of COVID-19 to 6th-semester medical students: experiences with a digital-only and a blended learning teaching approach using role-play and feedback.* GMS J Med Educ. 2021;38(7):Doc117. DOI: 10.3205/zma001513
